# The risk of central nodal metastasis based on prognostic factors of the differentiated thyroid carcinoma: a systematic review and meta-analysis study

**DOI:** 10.1007/s00405-023-07863-8

**Published:** 2023-02-10

**Authors:** Lamiaa Gomaa Hafez, Beshoy Effat Elkomos, Mahmoud Ahmed Mohamed El-Shafaei, Hesham Mohamed Ali Omran, Ahmed Saeed Saad

**Affiliations:** General Surgery Department, Faculty of Medicine, Ain Shams University, Tomanbia Street, Elzytoon, Cairo, Egypt

**Keywords:** Cancer thyroid, Predictive factors, Lymph node, Metastasis

## Abstract

**Background and aim:**

Despite improving the 10-year disease-free-survival, prophylactic central neck dissection (pCND) in differentiated thyroid carcinoma (DTC) should only be considered in patients with high risk factors for lymph node (LN) metastasis due to the increases in the risk of postoperative complications. Our aim was to identify the risk factors for central lymph node metastasis (CLNM) in DTC.

**Method:**

We searched PubMed, Scopus, Web of science, Cochrane library for eligible studies from inception to November 1, 2021 and a systematic review and meta-analysis were carried out to identify the risk factors for CLNM in DTC.

**Results:**

We included 41 studies with total of 27,741 patients in this study. The pooled results in this meta-analysis showed that these risk factors were significantly associated with CLNM: age < 45 years (odds ratio (OR) 1.64, 95% confidence interval (CI) 1.34–1.99, *p* < 0.00001), male sex (OR 1.73, 95% CI 1.54–1.93, *p* < 0.00001), multifocality (OR 1.87, 95% CI 1.59–2.19, *p* < 0.00001), bilateral disease (OR 1.43, 95% CI 1.15–1.78, *p* < 0.001), capsular invasion (OR 1.67, 95% CI 1.10–2.54, *p* < 0.02), lymphovascular invasion (OR 4.89, 95% CI 2.76–8.66, *p* < 0.00001) and extra-thyroidal extension (OR 2.43, 95% CI 1.97–3.00, *p* < 0.00001). In addition, young age (< 45 years), male sex, multifocality, and extra-thyroidal extension were significantly associated with large-volume CLNM in clinically N0 DTC patients. However, the presence of Hashimoto’s thyroiditis was not a predictors of large-volume CLNM.

**Conclusion:**

Young age (< 45 years), male sex, bilateral disease, multifocality, capsular invasion, lymphovascular invasion and extra-thyroidal extension are significantly associated with CLNM and pCND would be expected to have a higher yield in patients with these risk factors.

**Supplementary Information:**

The online version contains supplementary material available at 10.1007/s00405-023-07863-8.

## Introduction

Recently, the incidence of thyroid cancer has been shown to be increasing at the fastest rate among all malignancies globally [1, 2]. In 2020, 43,646 patients died from thyroid malignancy (27,740 females and 15,906 males) [3] and if this growth is maintained, by 2030, thyroid malignancy will be the fourth most common cancer [4].

Differentiated thyroid carcinoma (DTC), a term used to describe papillary thyroid cancer (PTC), follicular thyroid cancer (FTC) and Hürthle cell thyroid cancer (HTC), accounts for approximately 95% of all thyroid malignancies [5]. The expression ‘clinically lymph node-negative (cN0)’ is used to describe the patients without the clinical evidence of central lymph node metastasis (CLNM) on ultrasound or other imaging modalities preoperatively.

For clinically lymph node-positive patients, central neck dissection (CND) should be performed [6], while for cN0 patients, whether to perform prophylactic central neck dissection (pCND) remains controversial. Some studies have reported that bilateral prophylactic CND for staging of the neck in PTC, followed by personalized adjuvant radioiodine treatment, is associated with better 10-year disease-free-survival and loco-regional control of the disease, without increasing the risk of morbidity [7]. In contrast, other studies did not show a significant decrease in the risk of loco-regional control with pCND in patients with cN0 PTC [8]. Nevertheless, therapeutic CND based on some prognostic features associated with an increased risk of metastasis and recurrence (age, sex, multifocal disease, extra-thyroidal extension, capsular invasion and lymphovascular invasion) may be more reasonable than pCND [9–13]. Thus identifying risk factors of CLNM could guide surgeons to consider which cN0 thyroid malignancy patients require pCND. Our aim was to identify the risk factors for CLNM in differentiated thyroid malignancy.

## Patients and methods

### Search strategy

We searched PubMed, Web of science, Scopus, and the Cochrane Library for data from inception to November 1, 2021 using a combination of the following terms: “clinically node negative”, “Risk Factors” and “Thyroid Neoplasms”. All the studies were reviewed and evaluated by two authors (Hafez, L.G.& Elkomos, B.E.) according to the pre-defined eligibility criteria. We obtained full texts of the manuscripts found to be potentially eligible based on abstracts for full review.

### Inclusion and exclusion criteria

The eligible studies included the following: (1) randomized controlled trials and prospective or retrospective cohort studies; (2) target population were patients with DTC; (3) studies designating to detect the risk factors for LNM for cN0 as a primary aim; (4) studies providing a sufficient description of the methods and baseline characteristics; and (5) English language studies.

The following types of studies were excluded from our study: (1) unrelated or in vitro studies; (2) reviews, case reports and case series; (3) studies designed to analyse information from the United Network for Organ Sharing database; and (4) studies included patients with thyroid cancer other than DTC (e.g., medullary thyroid carcinoma, anaplastic thyroid carcinoma, lymphoma).

### Outcomes of interest

We assessed seven risk factors for LNM in CTC including age, sex, bilateral disease, multifocality, capsular invasion, lymphovascular invasion and extra-thyroidal extension as primary outcomes in this meta-analysis. In addition, our secondary outcomes were to detect the risk factors for large-volume LNM.

### Quality assessment and data extraction

A modification of the Newcastle–Ottawa scale was used to assess the quality of all cohort studies included in this meta-analysis [14]. This scale includes three domains: (1) selection of study groups (four points); (2) comparability of groups (two points); and (3) ascertainment of exposure and outcomes (three points). Only studies with seven or more scores were included.

We extracted data on study characteristics (author, year of publication and country where the study was conducted), patient characteristics (age and sex), follow up, tumor characteristics (size, bilateral disease, multifocality, capsular invasion, lymphovascular invasion, extra-thyroidal extension and recurrence). The data were extracted by two authors (Hafez, L.G.& Elkomos, B.E.) independently.

### Statistical analysis

This meta-analysis was conducted according to the Cochrane Handbook for Systematic Reviews of Interventions that is recommended by the Cochrane Collaboration. [15] The pooled odds ratios (ORs) and their corresponding 95% confidence intervals (CIs) were calculated with fixed effects models for all the risk factors. However, if there was moderate or considerable heterogeneity (*I*^2^ > 40), random effects models were used to solve the heterogeneity between studies. All calculations for the current meta-analysis were performed using the Review Manager 5.4 for Windows (Cochrane Collaboration, Oxford, United Kingdom).

### Assessment of publication bias and heterogeneity

Funnel plots were generated to visually inspect the publication bias. In addition, we used the statistical methods the Begg–Mazumdar rank correlation test and the Egger regression asymmetry test for detecting funnel plot asymmetry. Statistical heterogeneity was assessed with forest plots and the inconsistency statistic (*I*^2^). An *I*^2^ value of 40% or less corresponded to low heterogeneity. Statistical significance was considered at *p* < 0.05.

## Results

### Characteristics and quality assessment of eligible studies

**Fig. 1:**
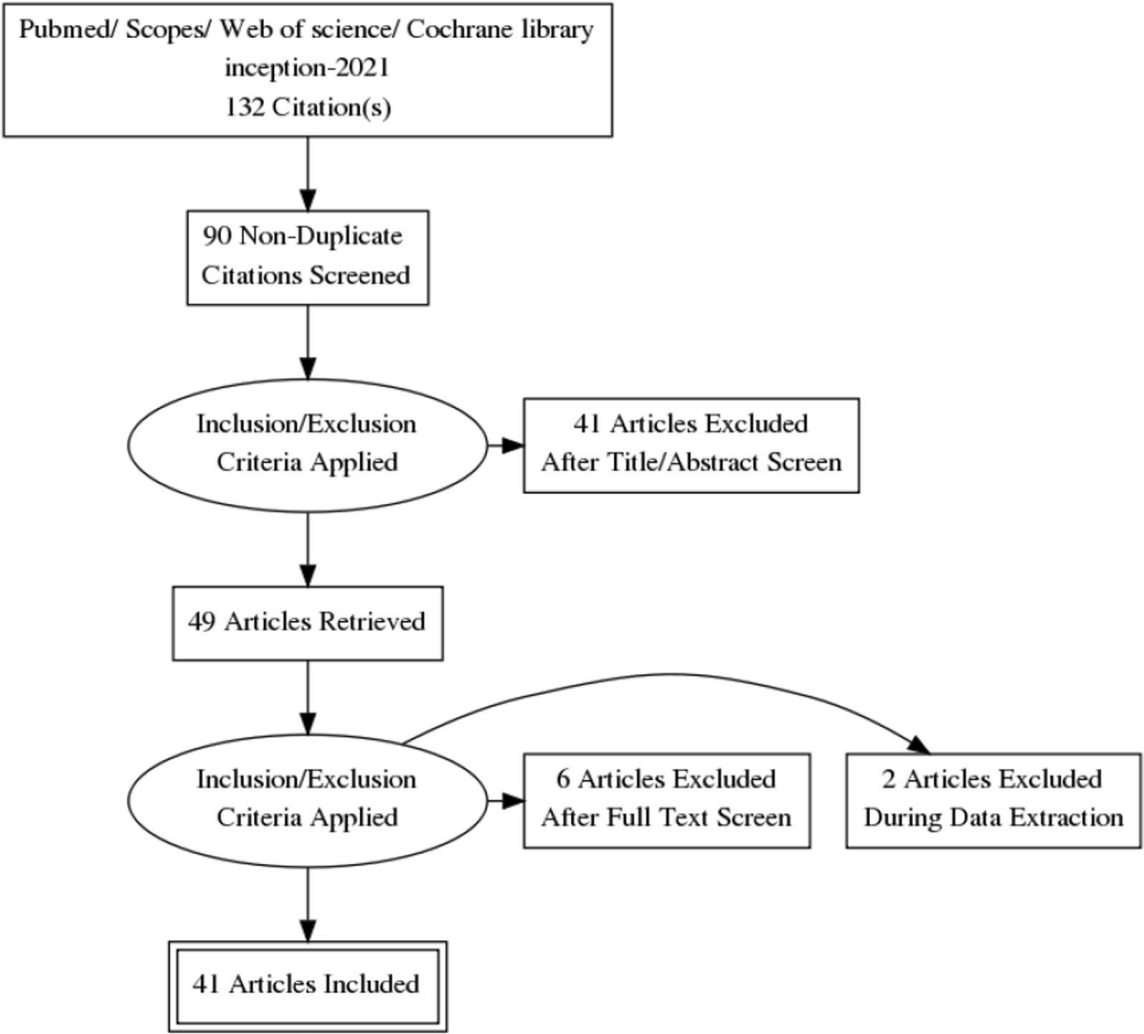
PRISMA flow diagram

As shown in Fig. 1, 132 articles were identified from the four databases using the following search string: “clinically node negative”, “Risk Factors” and “Thyroid Neoplasms”. After proper selection according to our eligibility criteria, 41 studies with 27,741 participants were included in the meta-analysis. These trials included 38 retrospective cohort studies and 3 prospective studies. However, none of the included studies were randomized studies.

Study characteristics [author, year of publication and country where the study was conducted], patient characteristics [age and sex], follow up time, tumor characteristics [size, bilateral disease, multifocality, capsular invasion, lymphovascular invasion, extra-thyroidal extension, and recurrence] were extracted from all the included studies (ESM Table 1). The quality assessment was conducted according to a modification of the Newcastle–Ottawa scale. Most of the cohort studies included in this analysis demonstrated sufficient quality with reasonable selection criteria, comparable patient characteristics, and adequate follow-up.

### Primary outcome

#### Age

**Fig. 2:**
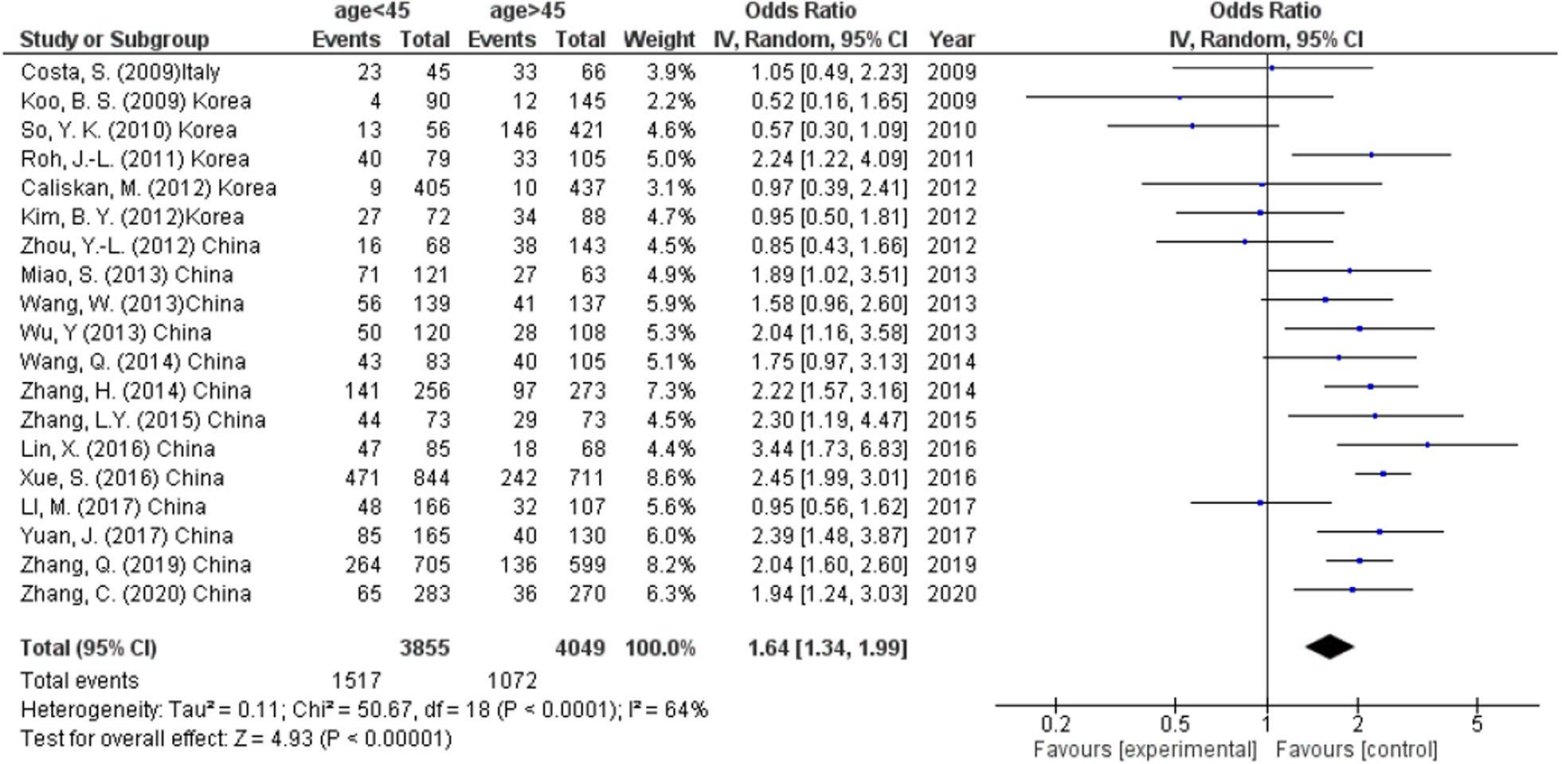
Age as a predictor of lymph node metastasis

According to 19 studies (7904 participants), the incidence of CLNM was significantly higher in the patient aged < 45 years (39.4%) than those age > 45 years (26.5%) (OR 1.64, 95% CI 1.34–1.99, *p* < 0.00001) (Fig. 2). A random effect was used to calculate odds ration because of high level heterogeneity (*I*^2^ = 64%, *p* < 0.0001).

#### Sex

**Fig. 3:**
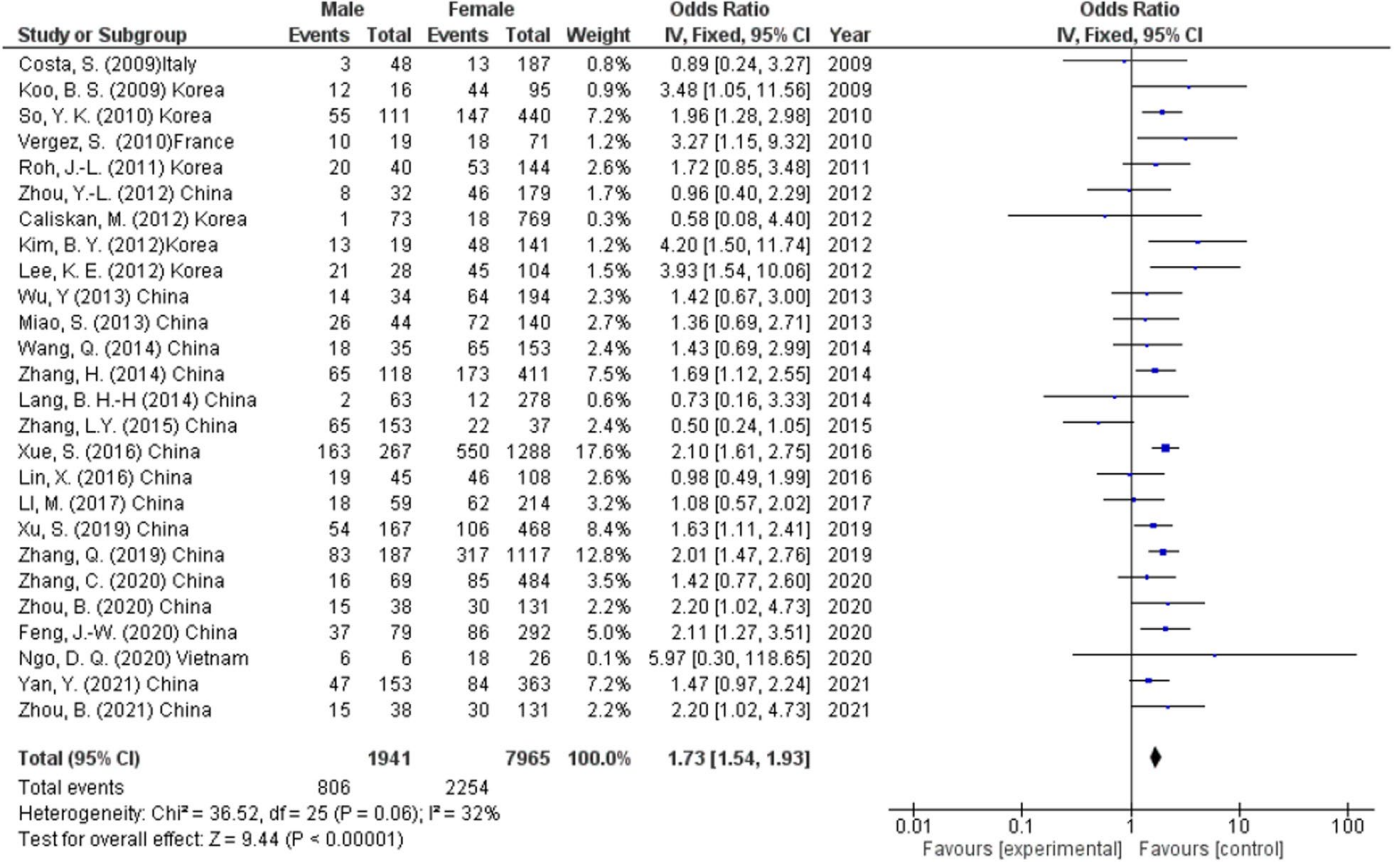
Sex as a predictor of lymph node metastasis

The incidence of CLNM was stated in 26 studies (9906 participants) and showed that the metastasis was more in male patients than female patients (41.5% and 28.3%, respectively) (OR 1.73, 95% CI 1.54–1.93, *p* < 0.00001) (Fig. 3). A fixed effect was used due to low level of heterogeneity (*I*^2^ = 32%, *p* < 0.06).

#### Multifocality

**Fig. 4:**
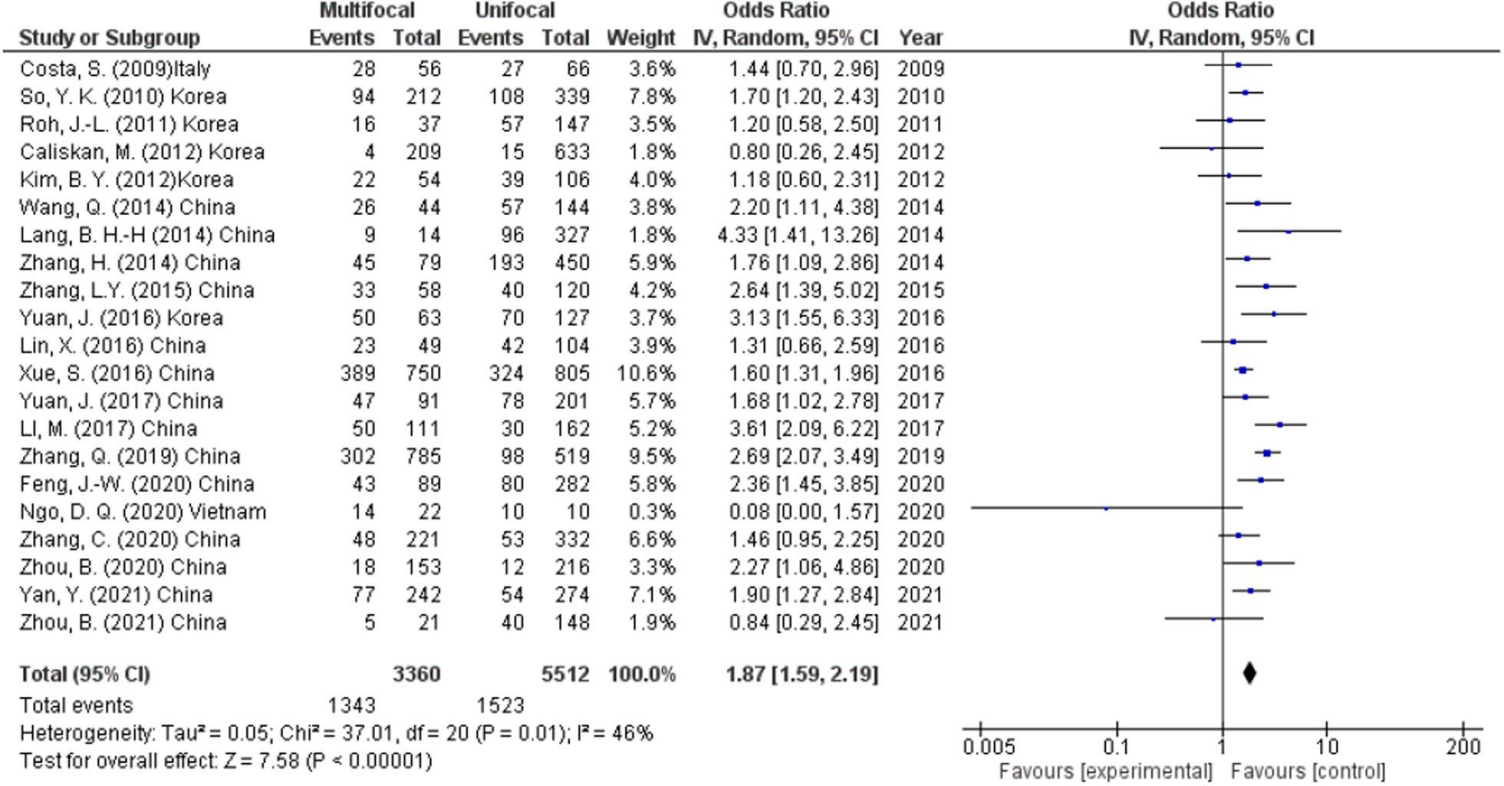
Multifocality as a predictor of lymph node metastasis

The relation between multifocality and LNM was reported in 21 studies (8872 participants). These studies revealed that the rate of CLNM was remarkably high in multifocal tumor than in unifocal tumor (40% and 27.6%, respectively) (OR 1.87, 95% CI 1.59–2.19, *p* < 0.00001) (Fig. 4). A random effect was used as heterogeneity was high (*I*^2^ = 46%, *p* < 0.01).

#### Bilateral disease

**Fig. 5:**
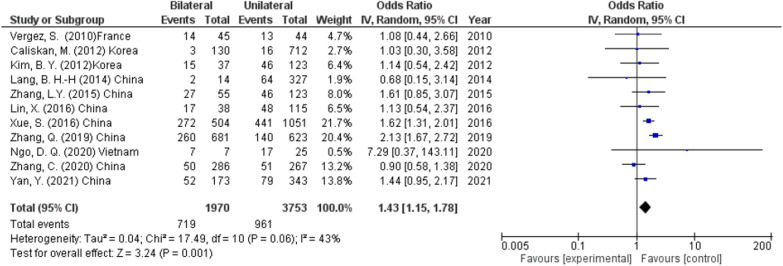
Bilateral disease as a predictor of lymph node metastasis

Eleven studies (5723 participants) reported the effect of the presence of bilateral thyroid tumor and the rate of CLNM. According to these studies, the patients with bilateral tumors showed a significantly higher incidence of CLNM than those with unilateral tumors (36.5% and 25.6%, respectively) (OR 1.43, 95% CI 1.15–1.78, *p* < 0.001) (Fig. 5). The heterogeneity was high (*I*^2^ = 43%, *p* < 0.06), so a random effect was used.

#### Capsular invasion

**Fig. 6:**
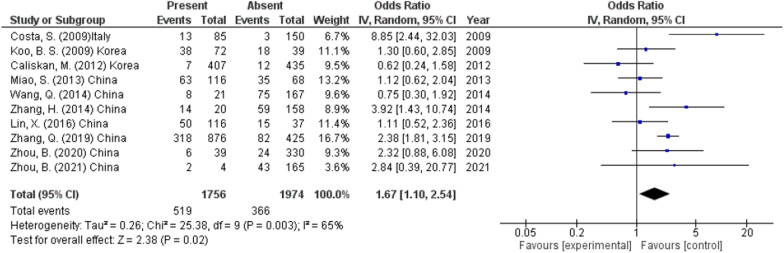
Capsular invasion as a predictor of lymph node metastasis

As reported by 10 studies (3730 participants), the presence of capsular invasion was a predictor of CLNM with a rate of 29.6% versus 18.5% in the absence of capsular invasion (OR 1.67, 95% CI 1.10–2.54, *p* < 0.02) (Fig. 6). A random effect was used as heterogeneity was high (*I*^2^ = 65%, *p* < 0.003).

#### Lymphovascular invasion

**Fig. 7:**
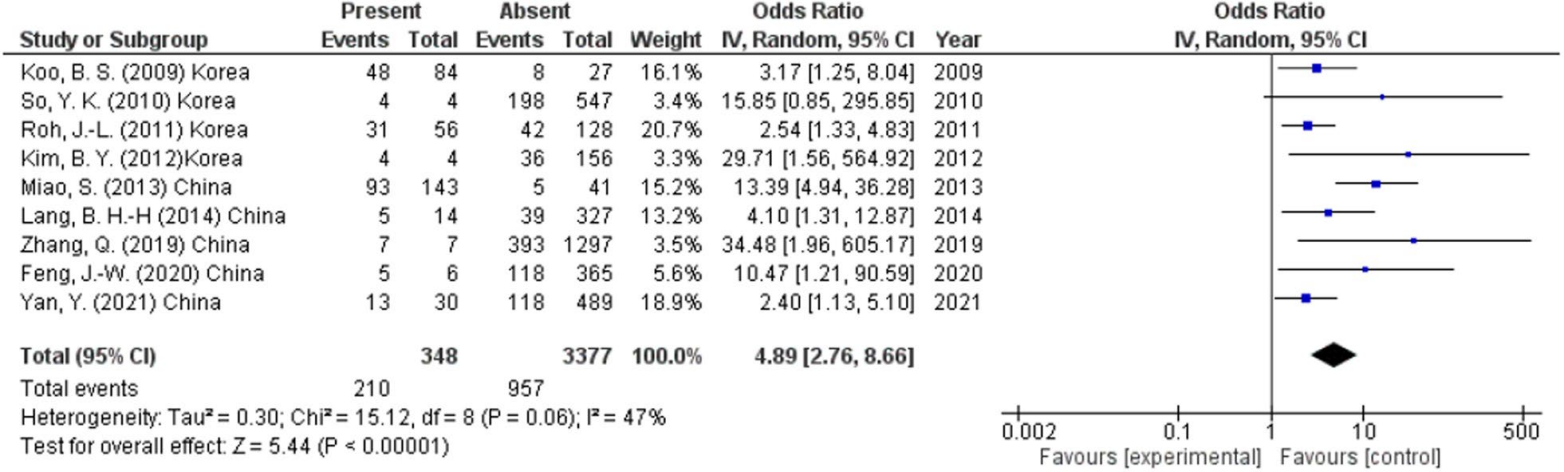
Lymphovascular invasion as a predictor of lymph node metastasis

According to the pooled ORs from 9 studies (3725 participants), CLNM was associated with the presence of LVI as the rate of CLNM was 60.3% versus 28.3% in the absence of lymphovascular invasion (OR 4.89, 95% CI 2.76–8.66, *p* < 0.00001) (Fig. 7). A random effect was used to solve heterogeneity (*I*^2^ = 47%, *p* < 0.06).

#### Extra-thyroidal extension

**Fig. 8:**
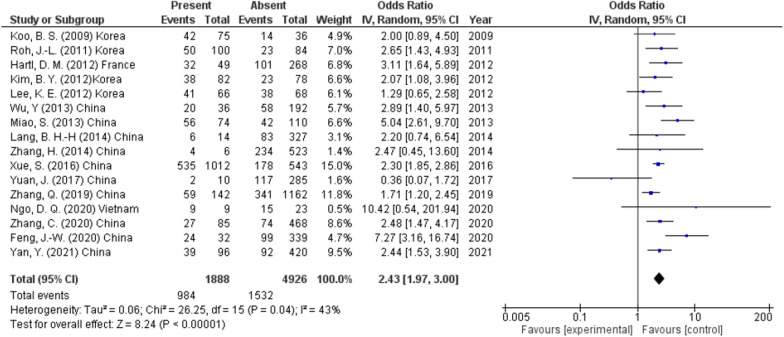
Extra-thyroidal extension as a predictor of lymph node metastasis

According to the pooled ORs in 16 studies (6814 participants), the incidence of recurrence after total thyroidectomy was significantly higher in the patients with extra-thyroidal extension than those without extra-thyroidal extension (52.1% and 31.1%, respectively) (OR 2.43, 95% CI 1.97–3.00, *p* < 0.00001) (Fig. 8). A random effect was used as heterogeneity was high (*I*^2^ = 43%, *p* < 0.04).

### Subgroup analysis

#### Countries (Asia, America and Europe)

A subgroup analysis was done to detect the risk factors of CLNM in clinically node negative patients according to the country. For example, according to the Chinese studies, young age (< 45 years), male sex, bilateral disease, multifocality, capsular invasion, lymphovascular invasion and extra-thyroidal extension were significantly associated with CLNM (ESM Table 2).

#### Secondary outcomes

According to ATA guidelines, [6] the presence of less than five pathological LNM (< 0.2 cm in the largest dimension) are classified as low-risk disease with recurrence risk < 5%. However, the presence of more than five metastatic lymph nodes (< 3 cm in the largest dimension) are classified as intermediate-risk disease with recurrence risk > 20%. Our secondary outcome was to detect the risk factors for large-volume lymph node metastasis (more than five metastatic lymph nodes) after total thyroidectomy. The risk factors for large- and small-volume lymph node metastases were reported in two studies (1311 Participants) and the pooled results showed that male gender, multifocality, and extra-thyroidal extension are significantly associated with large-volume CLNM in patients with DTC (Table 1).

**Table 1:** Risk factors for large-volume lymph node metastasis

Comparative group	Effect estimate [OR (95% CI)]	Heterogeneity	Test for overall effect	Group with high lymph node metastasis
Age	N/A	N/A	N/A	N/A
Sex (male)	1.96 [1.34, 2.87]	*I*^2^ = 0% (*p* = 0.66)	*Z* = 3.45 (*p* = 0.0006)	Male
Multifocality (presence)	1.79 [1.12, 2.86]	*I*^2^ = 0% (*p* = 0.93)	*Z* = 2.45 (*p* = 0.01)	Multifocal
Bilateral disease (presence)	N/A	N/A	N/A	N/A
Capsular invasion (presence)	N/A	N/A	N/A	N/A
Lynphovascular invasion (presence)	N/A	N/A	N/A	N/A
Extra-thyroidal extension (presence)	3.54 [2.28, 5.50]	*I*^2^ = 0% (*p* = 0.70)	*Z* = 5.61 (*p* < 0.00001)	Presence of extra-thyroidal extension
Hashimoto’s thyroiditis (presence)	0.83 [0.36, 1.89]	*I*^2^ = 53% (*p* = 0.15)	*Z* = 0.44 (*p* = 0.66)	None

#### Publication bias assessment

There was no evidence of publication bias. The funnel plot analysis demonstrated a symmetrical appearance, and the *p* values were greater than 0.05 for all comparisons according to the Begg–Mazumdar test and Eggers test.

## Discussion

The incidence of differentiated thyroid malignancy is rising worldwide [3]. According to the American Thyroid Association (ATA) guidelines, neck dissection is recommended for patients with cN1 DTC [6]. However, the sensitivity of ultrasound for detecting metastatic central neck lymph nodes preoperatively has been reported to range from 10.9 to 30%. This is because they are usually small and are obscured by the overlying thyroid gland [56–58]. In addition, 30–65% of patients with clinically node negative papillary thyroid microcarcinoma had CLNM (detected only on histopathology) [59, 60].

For these reasons, some studies have argued that prophylactic central neck dissection (pCND) allows staging of the cancer more accurately for evaluating the necessity of radioactive iodine therapy [61] and this will lead to a decrease in the local recurrence and improve disease-specific survival [62]. Moreover, the ATA guidelines also recommend prophylactic CND in patients with cN0 PTC, especially for advanced primary tumors (T3 or T4) [6]. On the other hand, prophylactic central neck dissection increases the risk of postoperative complications, mainly hypoparathyroidism and laryngeal nerve injury [63].

However, two factors are widely adopted and well-established. Firstly, there is an increased risk of complications in the second operation when the tumor recurs in the central compartment [64]. Secondly, lymph node metastasis has a strong association with recurrence after total thyroidectomy [65]. Therefore, some studies have recommended that prophylactic central neck dissection should only be considered in patients with high risk factors [61]. The pooled results in this meta-analysis showed that young age (< 45 years), male gender, bilateral disease, multifocality, capsular invasion, lymphovascular invasion and extra-thyroidal extension are significantly associated with CLNM in patients with clinically N0 DTC. In other words, prophylactic central neck dissection would be expected to have higher yield in patients with these risk factors.

To begin with the age, thyroid cancer rarely occurs in children under age of 15 years and the incidence of thyroid malignancy increases with age, peaking between the fifth and eighth decades [66]. However, the ATA risk stratification system does not include age as a predictor of recurrence. According to the pooled results from the included studies, the incidence of CLNM is significantly higher in the patient aged < 45 years than those aged > 45 years. Turning to the sex, despite being more common in females and this predominance has been explained by hormonal factors or biological changes that occur during pregnancy [67], this meta-analysis showed that the incidence of CLNM is higher in male sex.

According to Lu et al., multifocality may arise from intrathyroidal metastases from a single cancer cell clone or from multiple independent origins [68] and it can be seen in 18–87% of cases [69]. In this meta-analysis, it is a significant risk factor CLNM in cN0 thyroid malignancy. The Incidence of bilateral tumors is high and increases with the number of tumor foci in multifocal PTC [70, 71]. According to some studies, bilateral tumor is not more aggressive than unilateral disease with regards to histopathologic features, tumor stage and prognostic outcomes [72]. However, this meta-analysis showed that the patients with bilateral tumors have a significantly higher incidence of CLNM than those with unilateral tumors.

Tumor capsular invasion that is associated with the FTC has received more attention than in the PTC. This is because the differentiation between follicular adenomas and carcinomas depends on the demonstration of angioinvasion and/or tumor capsular invasion [73]. The presence of histologic capsule invasion is detected in approximately half of DTC, with a lower incidence in PTC (49.8%) than in FTC (73.5%) [74]. According to the pooled results of the included studies, our meta-analysis shows that the presence of capsular invasion is a predictor for CLNM.

Lymphovascular invasion in patients with thyroid cancer was described by Mete et al. by the presence of malignant cells in lymphovascular spaces or endothelial lining of lymphovascular cannels, invasion of malignant cells through a vessel wall and endothelium, or the presence of thrombus adherent to intravascular tumor [75]. The incidence of lymphovascular invasion in differentiated thyroid cancer ranges from 2 to 14% [76, 77]. Despite being a poor prognostic factor in different cancers [78, 79], lymphovascular invasion is not included into the staging systems for DTC. As reported by Pontius et al., the presence of lymphovascular invasion among patients with PTC is associated with significantly decreased survival [77]. In addition, this study showed that CLNM is significantly associated with the presence of lymphovascular invasion (60.3% versus 28.3% in the absence of lymphovascular invasion).

Extra-thyroidal extension has been defined as the growth of tumor outside the thyroid gland and into the nearby or surrounding tissues. For differentiated thyroid malignancies, the American Joint Committee on Cancer (AJCC) includes extra-thyroidal extension as part of the staging system. Stage T3 is defined as a “tumor greater than 4 cm limited to the thyroid or any tumor with minimal extra-thyroidal extension,” Stage T4a is defined as a “a tumor of any size that has grown beyond the thyroid capsule invading subcutaneous soft tissues, larynx, trachea, esophagus, or recurrent laryngeal nerve,” and stage T4b is defined as a “tumor of any size that encases the carotid artery or mediastinal vessels or invades the prevertebral fascia” [80]. As mentioned before, according to the ATA guidelines [6], prophylactic CND may be performed in patients with DTC with clinically uninvolved central neck lymph nodes, for advanced primary tumors (T3 or T4). However, these guidelines state that these are weak recommendations with poor-quality evidence. However, this meta-analysis showed that the incidence of recurrence after total thyroidectomy is significantly higher in the patients with extra-thyroidal extension than those without extra-thyroidal extension. In addition, Youngwirth et al. showed that extra-thyroidal extension is associated with a dramatic decrease in survival rate for patients with DTC [81].

The highest incident cases of thyroid cancer were reported in China and reached 41,511 cases in 2017 [82]. Our subgroup analysis according to the country where the study was conducted showed that, in China, age < 45 years, male sex, bilateral disease, multifocality, capsular invasion, lymphovascular invasion and extra-thyroidal extension are associated with LNM in clinically N0 DTC patients.

The recurrence rate after total thyroidectomy in large-volume LNM (more than five metastatic lymph nodes) was higher than that in small-volume LNM (less than or equal five metastatic lymph nodes) and the recurrence-free survival of large-volume LNM was dramatically poorer than that of small-volume LNM [49]. According to the pooled results from two of the included studies, young age (< 45 years), male gender, multifocality, and extra-thyroidal extension were significantly associated with large-volume LNM in clinically N0 DTC patients. However, the presence of Hashimoto’s thyroiditis was not a predictors of large-volume LNM.

To best of our knowledge, it is the first time the risk factors for large-volume LNM have been included in a meta-analysis. In addition, all the studies that reported the risk factors for LNM in clinically node negative for DTC were included in the study.

Nevertheless, we acknowledge some limitations in our study. Firstly, all the included studies were cohort studies and no randomized controlled trials could be found. Secondly, the heterogeneity in some results could not be explained in the subgroup analysis. Finally, only two of the included studies reported the risk factors for large-volume LNM. In other words, further studies are needed to better assess this point.

## Conclusion

Young age (< 45 years), male gender, bilateral disease, multifocality, capsular invasion, lymphovascular invasion and extra-thyroidal extension are associated with LNM in clinically N0 DTC patients and prophylactic central neck dissection would be expected to have higher yield in patients with these factors.

## Supplementary Information

Below is the link to the electronic supplementary material.

Supplementary file1 (PDF 530 KB)

Supplementary file2 (PDF 438 KB)

## Data Availability

The datasets generated during and/or analysed during the current study are available from the corresponding author on reasonable request. All data generated or analyzed during this study are included in this published article (and its supplementary information files).

## Abbreviations

DTC: Differentiated thyroid cancer
PTC: Papillary thyroid cancer
PTMC: Papillary thyroid microcarcinoma
FTC: Follicular thyroid cancer
HTC: Hürthle cell thyroid cancer
cN0: Clinically lymph node-negative
pCND: Prophylactic central neck dissection
CLNM: Central lymph node metastasis
LNM: Lymph node metastasis
ATA: American Thyroid Association
LVI: Lymphovascular invasion
