# Supplementary material for: The risk of central nodal metastasis based on prognostic factors of the differentiated thyroid carcinoma: a systematic review and meta-analysis study
Source: Eur Arch Otorhinolaryngol. 2023 Feb 10;280(6):2675–86. doi: 10.1007/s00405-023-07863-8 (PMC10175472; doi:10.1007/s00405-023-07863-8)
Supplement: Supplementary file 1 — Supplementary file1 (PDF 530 KB) [file 405_2023_7863_MOESM1_ESM.pdf]

Table 1: Basic data of the included studies.

| Studies: author, year, country | Study design | Study period | sample size | Age | Sex M:F | Follow up(months) | Tumor size | Multifocality | Bilaterallity | Extracapsular invasion | Extrathyroidal infiltration | Lymphovascular invasion | Pathology | Reccurence |
|---|---|---|---|---|---|---|---|---|---|---|---|---|---|---|
| **Koo, B. S. (2009) Korea [16]** | prospective cohort | 2005-2007 | 111 | 47.7 | 16/95 | N/A | 2– 48 mm | N/A | N/A | 72 | N/A | 84 | PTC | N/A |
| **COSTA, S. (2009) [17]** | retrospective cohort | 1994-2006 | 244 | 45 | 50/194 | N/A | <10mm 92 | 105 | N/A | N/A | N/A | N/A | PTC | 17 |
| **S. Vergez (2010)France [18]** | retrospective cohort | 2000-2006 | 90 | 45 ± 16 | 19/71 | 62 | N/A | N/A | N/A | N/A | N/A | N/A | PTC | N/A |
| **So, Y. K. (2010) Korea [19]** | retrospective cohort | 2005-2009 | 515 | 50.2 ± 9.2 | 111/440 | N/A | 6 ± 2 mm | 38.50% | 26.70% | 2.00% | 53.00% | 0.70% | PTMC | N/A |
| **Roh, J.-L. (2011) Korea [20]** | prospective cohort | 2005-2007 | 184 | 47 | 40/144 | N/A | 4-55 mm | 37 | 17 | N/A | 100 | 56 | PTC | N/A |
| **Zhou, Y.-L. (2012) China [21]** | retrospective cohort | 2010-2011 | 211 | 49±11 | 32/179 | N/A | 5.8±2.7 mm | 25 | N/A | N/A | 10 | N/A | PTMC | N/A |
| **Caliskan, M. (2012) [22]** | retrospective cohort | 2000-2005 | 842 | 46.27 ± 10.41 | 73/769 | ≥ 60 | <5mm 326 >5mm 516 | 209 | 130 | 407 | 23 | N/A | PTMC | N/A |
| **Kim, B. Y. (2012) [23]** | retrospective cohort | 2006-2010 | 160 | 47.3±11.8 | 19/141 | N/A | 6.8±2.1 mm | 54 | 37 | N/A | 82 | 4 | PTMC | N/A |
| **Hartl, D. M. (2012) France [24]** | retrospective cohort | 1997-2009 | 317 | 44 | 254/63 | 48 | 3-70 mm | N/A | N/A | N/A | 49 | N/A | PTC | N/A |
| **Lee, K. E. (2012) Korea [25]** | retrospective cohort | 2006-2007 | 161 | 20-73 | 36/125 | N/A | 2–60 mm | N/A | 42 | N/A | 93 | 13 | PTC | N/A |
| **Wu, Y (2013) China [26]** | retrospective cohort | 2007-2009 | 228 | 44 | 34/194 | N/A | N/A | N/A | N/A | N/A | N/A | N/A | PTC | N/A |
| **Wang, W. (2013) [27]** | retrospective cohort | 2008-2010 | 276 | 44.67 | 42/234 | N/A | N/A | N/A | 32 | N/A | N/A | N/A | PTC | N/A |
| **Miao, S. (2013) China [28]** | retrospective cohort | 2007-2009 | 184 | 39.8 | 44/140 | 24-50 | 2--68mm | N/A | N/A | N/A | N/A | N/A | PTC | N/A |
| **Lang, B. H.-H (2014) China [29]** | prospective cohort | 2003-2013 | 341 | 49.0±14.6 | 63/278 | N/A | 17.3±11.2mm | 105 | 66 | N/A | 89 | 44 | PTC | N/A |
| **Zhang, H. (2014) China [30]** | retrospective cohort | 2010-2012 | 529 | 44.5±11.1 | 118/411 | N/A | 16±11 mm | 79 | N/A | N/A | 6 | N/A | PTC | N/A |
| **Wang, Q. (2014) [31]** | retrospective cohort | N/A | 188 | 45 | 35/153 | N/A | <10mm 94 >10mm 94 | 44 | N/A | 21 | N/A | N/A | PTC | N/A |
| **Zhang L.Y. (2015) China [32]** | retrospective cohort | 2008-2010 | 178 | 46 | 37/141 | N/A | <6mm 95 >6mm 83 | 58 | 55 | 20 | N/A | N/A | PTMC | N/A |
| **Park, K.M. (2015) [33]** | retrospective cohort | 2009-2013 | 264 | 48.4 ±11.9 | 42/222 | N/A | 10.6± 5.7mm | 116 | N/A | N/A | 165 | N/A | PTC | |
| **Gao, Y. (2015) China [34]** | retrospective cross sectional | 2013 | 163 | 45.4±10.6 | 36/127 | N/A | 7.1±1.8 mm | 32 | N/A | 77 | 36 | 69 | PTMC | N/A |
| **Yuan, J. (2016) Korea [35]** | retrospective cohort | 2014-2015 | 190 | 41±11.6 | 61-129 | N/A | >20mm 83 <20mm 107 | 63 | 55 | 60 | 19 | N/A | PTC | N/A |
| **Xue, S. (2016) China [36]** | retrospective cohort | 2003-2008 | 1555 | 43.25 ± 10.38 | 267 / 1388 | N/A | 1-80 mm | N/A | 504 | N/A | N/A | N/A | PTC | N/A |
| **Lin, X. (2016) China [37]** | retrospective cohort | 2014 | 153 | 3.1±13.1 | 45/108 | N/A | >20mm 33 <20mm 120 | 49 | N/A | 37 | N/A | N/A | DTC | N/A |
| **Kim, S. K. (2016) Korea [8]** | retrospective cohort | 1997- 2015 | 11569 | >18 | N/A | 62.6 | N/A | N/A | N/A | N/A | N/A | N/A | PTC | N/A |
| **Yuan, J. (2017) China [38]** | retrospective cohort | 2014-2015 | 295 | 43.0 ± 12.3 | 89/206 | N/A | >5mm 107 <5mm 188 | 91 | 76 | 43 | 10 | 4 | PTC | N/A |
| **M. LI (2017) China [39]** | retrospective cohort | 2014-2016 | 273 | 43.7 ±13.1 | 59/214 | N/A | >3m 243 <3mm 30 | 111 | 61 | 17 | N/A | N/A | PTMC | N/A |
| **Sessa, L. (2017) Italy [40]** | prospective cohort | 2008-2012 | 182 | 42.6 ± 14.5 | 38/148 | N/A | 14.7 ± 9.2 | 128 | 97 | 57 | N/A | 27 | PTC | N/A |
| **Calò, P. G. (2017) Italy [41]** | retrospective cohort | 2008-2012 | 258 | 44.4 ±11.8 | 202/56 | 72.5 ±8.4 | N/A | 99 | N/A | N/A | 60 | 23 | DTC | 5 |
| **An, C. (2017) China [42]** | retrospective cohort | 2006-2013 | 138 | 18-71 | 33/108 | 36 | 1-50 mm | 36 | N/A | N/A | N/A | N/A | PTC | 1 |
| **Han, Z. (2018) [43]** | retrospective cohort | 2003-2008 | 151 | 37.41± 9.49 | 32/119 | N/A | 12- 81 mm | 62 | 46 | N/A | N/A | N/A | PTC | N/A |
| **Ryu, Y. J. (2018) Korea [44]** | retrospective cohort | 2004-2008 | 1406 | 47 | 190/1216 | 107 | >10 mm 431 ,>20mm 145 | 324 | 294 | N/A | N/A | 27 | PTC | 37 |
| **Zhang, Q. (2019) China [45]** | retrospective cohort | 2013-2016 | 1304 | 44.9 ± 9.32 | 187/1117 | N/A | 5.4 ± 2.1 mm | 785 | 681 | 879 | 142 | 7 | PTMC | N/A |
| **Xu, S. (2019) China [46]** | retrospective cohort | 2018-2019 | 635 | 42.6 ± 12.4 | 467/168 | N/A | 9.0 ± 4.8 mm | N/A | N/A | N/A | N/A | N/A | PTC | N/A |
| **Ryu, Y. J. (2019) Korea [47]** | retrospective cohort | 2004-2012 | 1082 | 46 | 213/869 | 78 | >10 mm 430 <10mm 652 | 331 | 424 | N/A | N/A | 23 | PTC | 62 |
| **Ngo, D. Q. (2020) Vietnam [48]** | retrospective cohort | 2015-2019 | 32 | 15.5 | 6--26 | N/A | <10mm 15 >10mm 17 | 10 | 7 | N/A | 9 | N/A | PTC | N/A |
| **Shen, G. (2020) China [49]** | retrospective cohort | 2009-2015 | 947 | 44.0±10.0 | 248/699 | N/A | 7.5±2.4 cm | 84 | N/A | N/A | 91 | N/A | PTMC | N/A |
| **Zhang, C. (2020)China [50]** | retrospective cohort | 2015-2017 | 553 | 45.24±8.56 | 69/484 | N/A | N/A | N/A | N/A | N/A | N/A | N/A | PTMC | N/A |
| **Zhou, B. (2020) China [51]** | retrospective cohort | 2013-2016 | 369 | N/A | 75/294 | N/A | <10 mm 312 | 153 | N/A | 39 | N/A | N/A | PTC | N/A |
| **Feng, J.-W. (2020) China [52]** | retrospective cohort | 2010-2018 | 371 | 45.7±11.7 | 79/292 | 47 | 6.7±2.4 cm | 89 | N/A | N/A | 32 | 6 | PTMC | 14 |
| **Yan, Y. (2021) China [53]** | retrospective cohort | 2017-2020 | 516 | N/A | N/A | N/A | N/A | N/A | N/A | N/A | N/A | N/A | PTC | N/A |
| **Zhou, B. (2021) China [54]** | retrospective cohort | 2019-2020 | 212 | 18-55 171, >55 41 | 49/163 | N/A | <5mm 45 ,5mm<n<10 mm 103,>10mm 35 | 29 | N/A | 23 | N/A | N/A | PTC | N/A |
| **Huang, J. (2021) China [55]** | retrospective cohort | 2017-2019 | 364 | 41.02±11.324 | 235/129 | N/A | <10mm 232 >10mm 132 | 121 | 90 | 87 | 56 | N/A | PTC | N/A |

(*) The results are presented as means and standard deviation.

(**) The results are presented as median and range
