# Supplementary material for: The risk of central nodal metastasis based on prognostic factors of the differentiated thyroid carcinoma: a systematic review and meta-analysis study
Source: Eur Arch Otorhinolaryngol. 2023 Feb 10;280(6):2675–86. doi: 10.1007/s00405-023-07863-8 (PMC10175472; doi:10.1007/s00405-023-07863-8)
Supplement: Supplementary file 2 — Supplementary file2 (PDF 438 KB) [file 405_2023_7863_MOESM2_ESM.pdf]

Table 2: Risk factors according to the country.

Subgroup	Country	Studies (n)	Patient (n)	Effect Estimate [OR (95% CI)]	Heterogeneity	Test for Overall Effect	Group with high LNM
Age	China	13	5895	1.94 [1.64, 2.30]	I^2 = 45% (P = 0.04)	Z = 7.79 (P < 0.00001)	Age < 45
	Korea	5	1898	0.96 [0.55, 1.68]	I^2 = 63% (P = 0.03)	Z = 0.15 (P = 0.88)	None
	Others	1	111	N/A	N/A	N/A	None
Sex	China	18	7569	1.55 [1.31, 1.83]	I^2 = 34% (P = 0.08)	Z = 5.21 (P < 0.00001)	Male
	Korea	6	1980	2.30 [1.59, 3.32]	I^2 = 16% (P = 0.31)	Z = 4.45 (P < 0.00001)	Male
	Others	3	357	2.09 [0.76, 5.78]	I^2 = 29% (P = 0.24)	Z = 1.43 (P = 0.15)	None
multifocality	China	14	6791	2.01 [1.68, 2.40]	I^2 = 46% (P = 0.03)	Z = 7.69 (P < 0.00001)	Multifocal
	Korea	5	1927	1.56 [1.08, 2.25]	I^2 = 38% (P = 0.17)	Z = 2.36 (P = 0.02)	Multifocal
	Others	2	154	0.50 [0.03, 7.58]	I^2 = 71% (P = 0.06)	Z = 0.50 (P = 0.62)	None
Bilaterality	China	7	4600	1.46 [1.14, 1.88]	I^2 = 59% (P = 0.02)	Z = 2.97 (P = 0.003)	Bilateral
	Korea	2	1002	1.11 [0.58, 2.11]	I^2 = 0% (P = 0.89)	Z = 0.32 (P = 0.75)	None
	Others	2	121	1.62 [0.35, 7.52]	I^2 = 31% (P = 0.23)	Z = 0.62 (P = 0.54)	None
Capsular invasion incasion	China	7	2542	1.69 [1.10, 2.59]	I^2 = 56% (P = 0.004)	Z = 2.42 (P = 0.02)	Presence of C I
	Korea	2	953	0.94 [0.45, 1.95]	I^2 = 30%(P = 0.23)	Z = 0.16 (P = 0.87)	None
	Others	1	235	N/A	N/A	N/A	N/A
Lymphovascular invasio	China	5	2719	6.30 [2.54, 15.61]	I^2 = 59% (P = 0.05)	Z = 3.98 (P < 0.0001)	Presence of LV I
	Korea	4	1006	3.42 [1.73, 6.74]	I^2 = 22%(P = 0.28)	Z = 3.54 (P = 0.0004)	
	Others	0	0	N/A	N/A	N/A	N/A
Extra thyroid extension	China	10	5876	2.55 [1.91, 3.40]	I^2 = 58% (P = 0.01)	Z = 6.38 (P < 0.00001)	Extra thyroid extension
	Korea	4	589	1.98 [1.40, 2.78]	I^2 = 0% (P = 0.51)	Z = 3.91 (P < 0.0001)	
	Others	2	349	3.28 [1.76, 6.13]	I^2 = 0% (P = 0.43)	Z = 3.74 (P = 0.0002)	
